# The association of angiogenic factors and chronic kidney disease

**DOI:** 10.1186/s12882-018-0909-2

**Published:** 2018-05-21

**Authors:** Christopher E. Anderson, L. Lee Hamm, Gem Batuman, Damodar R. Kumbala, Chung-Shiuan Chen, Swapna G. Kallu, Ravi Siriki, Shilpa Gadde, Myra A. Kleinpeter, N. Kevin Krane, Eric E. Simon, Jiang He, Jing Chen

**Affiliations:** 1 Department of Epidemiology, Tulane University School of Public Health and Tropical Medicine, 1440 Canal Street, room 1504, New Orleans, LA 70112 USA; 2 Department of Medicine, Tulane University School of Medicine, 1430 Tulane Avenue SL45, New Orleans, LA 70112 USA; 3 Tulane Hypertension and Renal Center of Excellence, Tulane University School of Medicine, New Orleans, LA USA; 4 Department of Nephrology, Ochsner Health System, Ochsner Medical Center, 1514 Jefferson Highway, New Orleans, LA 70121 USA

**Keywords:** Angiopoietin-1, Vascular endothelial growth factor-a, Pentraxin-3, Chronic kidney disease

## Abstract

**Background:**

There are limited data on the associations of circulating angiogenic factors with chronic kidney disease (CKD). We investigate the associations of circulating vascular endothelial growth factor (VEGF)-A, angiopoietin-1, angiopoietin-1/VEGF-A ratio, VEGF receptor 1 (VEGFR-1), VEGFR-2, and pentraxin-3 with CKD.

**Methods:**

We recruited 201 patients with CKD and 201 community controls without CKD from the greater New Orleans area. CKD was defined as estimated glomerular filtration rate (eGFR) < 60 mL/min/1.73 m^2^ or presence of albuminuria. Multivariable quantile and logistic regression models were used to examine the relationship between angiogenesis-related factors and CKD adjusting for confounding factors.

**Results:**

After adjusting for covariables including traditional cardiovascular disease (CVD) risk factors, C-reactive protein, and history of CVD, the medians (interquartile range) were 133.08 (90.39, 204.15) in patients with CKD vs. 114.17 (72.45, 170.32) pg/mL in controls without CKD (*p* = 0.002 for group difference) for VEGF-A; 3951.2 (2471.9, 6656.6) vs. 4270.5 (2763.7, 6537.2) pg/mL (*p* = 0.70) for angiopoietin-1; 25.87 (18.09, 47.90) vs. 36.55 (25.71, 61.10) (*p* = 0.0001) for angiopoietin-1/VEGF-A ratio; 147.81 (122.94, 168.79) vs. 144.16 (123.74, 168.05) ng/mL (*p* = 0.25) for VEGFR-1; 26.20 (22.67, 29.92) vs. 26.28 (23.10, 29.69) ng/mL (*p* = 0.31) for VEGFR-2; and 1.01 (0.79, 1.49)vs. 0.89 (0.58, 1.18) ng/mL (*p* = 0.01) for pentraxin-3, respectively. In addition, an elevated VEGF-A level and decreased angiopoietin-1/VEGF-A ratio were associated with increased odds of CKD.

**Conclusions:**

These data indicate that plasma VEGF-A and pentraxin-3 levels were increased and the angiopoietin-1/VEGF-A ratio was decreased in patients with CKD. Future prospective studies are warranted to examine whether angiogenic factors play a role in progression of CKD.

**Electronic supplementary material:**

The online version of this article (10.1186/s12882-018-0909-2) contains supplementary material, which is available to authorized users.

## Background

Chronic Kidney Disease (CKD) is a highly prevalent disease, affecting over 26.3 million adults in the US alone and 497.5 million in the world [1, 2]. CKD has been associated with increased risks of end-stage renal disease (ESRD), cardiovascular disease (CVD), and premature death [3, 4]. Traditional risk factors only partially explain excess risk of CKD and associated ESRD and CVD in the general populations [5]. Identification of novel risk factors for CKD will further the understanding of CKD pathogenesis and provide additional targets for therapies [6, 7].

Animal studies have suggested the involvement of an imbalance of angiogenic factors in the pathogenesis of kidney disease [8–14]. In experimental studies, treatment with angiopoietin-1 reduced tubular injury in unilateral ureteral obstruction [15], decreased albuminuria in streptozotocin-induced type-1 diabetes [16], and stabilized peritubular capillaries in folic acid nephropathy, though this was accompanied by profibrotic and inflammatory effects [17]. Deletion of angiopoietin-1 coupled with microvascular stress resulted in organ damage, accelerated angiogenesis and fibrosis [18].

The findings regarding the association of angiogenic factors and CKD in human are somewhat inconsistent likely due to diversities in sample size, study population, sources of angiogenic factors, and covariables used in the analyses. It was recently reported that vascular endothelial growth factor (VEGF)-A predicted CKD progression in diabetic patients in a small cohort study [19]. However, another study suggested VEGF expression was reduced in the biopsied kidney tissue from patients with diabetic nephropathy [20]. Elevated soluble VEGF receptor-1 (sVEGFR-1) and reduced VEGFR-2 were associated with mortality in dialysis patients [21, 22]. Angiopoietin-1 mediates migration, adhesion, and survival of endothelial cells, and co-expression of angiopoietin-1 and VEGF enhances angiogenesis [23]. Decreased angiopoietin-1 and increased angiopoietin-2 levels have been identified in patients with CKD [24, 25]. Associations of angiopoietin-2 [24–27] and angiopoietin-1 [27] with subclinical CVD have been reported in CKD. Angiopoietin-2 was found to be associated with increased mortality among CKD patients [24]. Pentraxin-3 can bind fibroblast growth factor-2 (FGF2) and act as a FGF2 antagonist to inhibit FGF2-dependent angiogenesis [26].

This study aims to examine the association between multiple circulating angiogenic factors and CKD in a larger pre-dialysis CKD population.

## Methods

### Study participants

Two hundred one patients with CKD and 201 controls without CKD were recruited between 2007 and 2010 in the greater New Orleans area. The patients with CKD were recruited from nephrology and internal medicine clinics by trained research staff in the study area. These patients were between 21 and 74 years of age. All of the eligible CKD cases identified through the referral clinics were invited to participate. CKD was defined as an eGFR < 60 ml/min/1.73m^2^ or presence of albuminuria (> 30 mg/24-h). Exclusion criteria were a history of chronic dialysis, acute kidney injury, kidney transplant, pregnancy, immunotherapy in the preceding 6 months, chemotherapy in the preceding 2 years, HIV or AIDS, being unable or unwilling to provide informed consent, and participating in a current clinical trial that might have an impact on CKD. Controls were recruited through mass mailing to residents between 21 and 74 years of age residing in the same area, determined by zip code. Control eligibility for participation was assessed by a clinic screening visit.

The Institutional Review Board of Tulane University approved the conduct of this study, and written informed consent was obtained at the screening visit from all participants.

### Data collection

Trained staff administered a questionnaire at a clinical visit to obtain demographic information, lifestyle factors (e.g., cigarette smoking, alcohol consumption, and physical activity), medical history (CVD, diabetes, hypercholesterolemia and hypertension), and the use of medications including aspirin and antihypertensive, hypoglycemic, and lipid-lowering agents.

Three blood pressure (BP) measurements were obtained by trained and certified staff at a clinical visit according to a standard protocol adapted from American Heart Association recommendations [27]. BP was measured using a standard mercury sphygmomanometer, with one of four cuff sizes based on the patient’s arm circumference, on the patient in a seated position and after 5 min of rest. Height and weight were measured twice in patients in lightweight indoor clothing without shoes during the clinical visit and were used to calculate body mass index (BMI).

An overnight fasting blood sample was collected to measure glucose, creatinine, cholesterol, triglycerides, and angiogenesis-related biomarkers. Samples were stored at − 80 degrees Celsius (− 80 °C). All samples were measured after being stored for less than 5 years. All of the biomarkers have been previously reported to be stable when stored at − 80 °C [28, 29]. Multiple freeze thaw cycles can increase concentrations of VEGF-A [30]. Biomarkers were measured after the first thaw of the samples to minimize the opportunity for freeze-thaw cycle related changes. Serum creatinine was measured using the Roche enzymatic method (Hoffman-La Roche, Basel, Switzerland). eGFR was estimated based on serum creatinine (SCr), sex, age, and race using the CKD-Epi equation [31]. A 24-h urinary sample was collected to measure creatinine and albumin. Serum cholesterol and triglyceride levels were assayed using an enzymatic procedure on the Hitachi 902 automatic analyzer (Roche Diagnostics, Indianapolis, IN, USA). Serum glucose was measured using a hexokinase enzymatic method (Roche Diagnostics, Indianapolis, IN, USA). Urinary concentrations of albumin and creatinine were measured with a DCA 2000 Analyzer (Bayer AG, Leverkusen, Germany). Plasma VEGF-A, sVEGFR-1, VEGFR-2, and angiopoietin-1 were measured using a sandwich immunoassay on a Meso Scale Discovery Instrument (Meso Scale Diagnostics, LLC., Rockville, MD, USA). Plasma pentraxin-3 was measured by the ELISA assay from R & D Systems (Minneapolis, MN, USA). A stringent quality control process was applied in all laboratory tests. All biomarkers were measured in duplicate, with inter-assay coefficients of variation 23.4% for VEGF-A, 2.4% for sVEGFR-1, 2.6% for VEGFR-2, 10.13% for angiopoietin-1, and 7.1% for pentraxin-3, respectively. All laboratory measures were conducted at the Laboratory for Clinical Biochemistry Research, the University of Vermont.

### Statistical analysis

Characteristics of CKD cases and non-CKD controls were compared using Chi-square tests for categorical variables and t-tests for the continuous variables. Medians and interquartile ranges of the angiogenesis-related biomarkers were calculated for the CKD patients and controls and the differences were compared using the Mann-Whitney test [32]. Quantile regression was used to obtain adjusted-medians and interquartile ranges [33]. The Wald test was used to assess differences in the adjusted medians between CKD patients and controls [33]. The covariates included in the multivariable quantile regression model were age, race, gender, current cigarette smoking, weekly alcohol consumption, physical activity, BMI, LDL-cholesterol, HDL-cholesterol, C-reactive protein, fasting plasma glucose, systolic BP, self-reported history of CVD, and use of aspirin and hypoglycemic, antihypertensive, and lipid-lowering agents.

Multivariable logistic regression models were used to assess adjusted-odds ratios comparing the highest tertile of angiogenesis-related biomarkers to the lower two tertiles between CKD patients and the controls (except for angiopoietin-1/VEGF-A ratio, in which the lowest tertile was compared to the higher two). The same panel of covariates used in the multivariable quantile regression was included in the multivariable logistic regression models. This analysis was also performed stratified by diabetes status.

Associations between the angiogenesis biomarkers and stage of CKD were assessed using polytomous logistic regression and quantile regression. Stages 4 and 5 were combined due to small sample size in each category.

A sensitivity analysis was performed among CKD participants in which the medians of the angiogenesis-related biomarkers were compared between diabetic CKD cases and non-diabetic CKD cases.

## Results

Characteristics of the study participants are presented in Table 1. Those with CKD were older, more likely to report a history of CVD, hypertension, diabetes, and dyslipidemia, and to have self-reported use of antihypertensive, hypoglycemic, lipid-lowering agents, or aspirin, and were less likely to drink alcohol, have a high-school education, or be physically active. Those with CKD had higher average BMI, systolic BP, and fasting glucose, but lower LDL-cholesterol and HDL-cholesterol.

**Table 1:** Characteristics of 201 patients with chronic kidney disease and 201 controls without chronic kidney disease

	CKD Patients (*N* = 201)	Non-CKD (*N* = 201)	*P*-value
Age, years	55.9 ± 9.9	52.5 ± 10.0	< 0.001
Male, %	55.2	45.3	0.06
African-American, %	60.7	51.2	0.06
Current cigarette smoking, %	53.7	48.8	0.32
Alcohol consumption, %	27.9	59.2	< 0.001
Physical activity ≥twice/week, %	53.0	72.9	< 0.001
High school education, %	58.5	81.6	< 0.001
History of CVD, %	43.7	7.0	< 0.001
History of hypertension, %	88.1	23.9	< 0.001
History of diabetes, %	49.3	5.5	< 0.001
History of hypercholesterolemia, %	65.7	30.9	< 0.001
Use of antihypertensive agents, %	79.6	15.4	< 0.001
Use of hypoglycemic agents, %	34.3	3.0	< 0.001
Use of lipid lowering agents, %	21.9	8.9	< 0.001
Use of aspirin, %	34.8	8.5	< 0.001
BMI, kg/m^2^	32.2 ± 7.8	28.9 ± 6.4	< 0.001
Systolic BP, mm Hg	132.2 ± 21.0	122.0 ± 14.7	< 0.001
Diastolic BP, mm Hg	77.2 ± 13.5	77.6 ± 9.4	0.77
LDL-cholesterol, mg/dL	101.8 ± 47.3	118.2 ± 30.2	< 0.001
HDL-cholesterol, mg/dL	50.3 ± 15.6	57.7 ± 18.0	< 0.001
Fasting plasma glucose, mg/dL	119.9 ± 46.8	103.4 ± 35.4	< 0.001
C-reactive protein mg/L	5.3 ± 11.7	4.1 ± 8.4	0.26
eGFR, mL/min/1.73 m^2^	43.3 ± 19.3	96.7 ± 16.8	< 0.001
Urinary albumin, mg/24 h	74.5 (12.3, 417.4)	5.9 (4.1, 11.4)	< 0.001

Categorical variables are presented as percentages while continuous variables are presented as mean and standard deviation or median and interquartile range

*LDL* low-density lipoprotein, *HDL* high-density lipoprotein, *eGFR* estimated glomerular filtration rate, *BMI* body mass index, *BP* blood pressure, *CVD* cardiovascular disease

The age-gender-race adjusted and multivariable-adjusted medians of the angiogenesis-related biomarkers are presented in Table 2. After adjusting for potential confounding factors, the medians of VEGF-A and pentraxin-3 were significantly higher in CKD patients than controls while that of the angiopoietin-1/VEGF-A ratio was significantly lower in CKD patients than in controls. The medians of angiopoietin-1, VEGFR-1, and VEGFR-2 were not significantly different between CKD patients and controls.

**Table 2:** Angiogenesis-related factors according to chronic kidney disease status

	Age-gender-race-adjusted median (IQR)			Multivariable-adjusted median (IQR)^a^		
	CKD patients (*n* = 201)	Non-CKD controls (*n* = 201)	*P*	CKD patients (*n* = 201)	Non-CKD controls (*n* = 201)	*P*
VEGF-A, pg/mL	132.6 (90.4, 199.0)	112.5 (71.9, 166.8)	0.13	133.08 (90.39, 204.15)	114.17 (72.45, 170.32)	0.002
Angiopoeitin-1, pg/mL	3957.1 (2471.9, 6602.1)	4269.1 (2668.5, 6501.9)	0.34	3951.2 (2471.9, 6656.6)	4270.5 (2763.7, 6537.2)	0.70
Angiopoeitin-1/VEGF-A	25.80 (18.09, 47.90)	36.69 (25.71, 61.10)	< 0.001	25.87 (18.09, 47.90)	36.55 (25.71, 61.10)	< 0.001
VEGFR-1, ng/mL	148.0 (122.9, 167.9)	144.2 (123.7, 168.0)	0.92	147.81 (122.94, 168.79)	144.16 (123.74, 168.05)	0.25
VEGFR-2, ng/mL	26.1 (22.7, 29.9)	26.4 (23.1, 29.7)	0.79	26.20 (22.67, 29.92)	26.28 (23.10, 29.69)	0.31
Pentraxin-3, ng/mL	1.02 (0.79, 1.48)	0.86 (0.58, 1.17)	0.01	1.01 (0.79, 1.49)	0.89 (0.58, 1.18)	0.01

*VEGF-A* vascular endothelial growth factor A; VEGFR-1 = vascular endothelial growth factor receptor 1; VEGFR-2 = vascular endothelial growth factor receptor 2; CKD = chronic kidney disease; IQR = interquartile range

^a^Multivariable adjusted model adjusted for age, race, gender, current cigarette smoking, weekly alcohol consumption, physical activity ≥twice/week, BMI, LDL-cholesterol, HDL-cholesterol, C-reactive protein, fasting plasma glucose, systolic BP, use of aspirin or lipid-lowering, antihypertensive, or antidiabetic medications, and history of CVD

In multivariable logistic regression analysis adjusting for important confounding factors, the odds of CKD were more than doubled for subjects with the highest tertile of VEGF-A compared to those in the lower two tertiles (Table 3). In addition, the odds of CKD were more than three times greater for subjects with the lowest tertile of the angiopoietin-1/VEGF-A ratio compared to those in the higher two tertiles. The levels of angiopoietin-1, VEGFR-1, VEGFR-2 and pentraxin-3 were not significantly associated with increased odds of CKD in the multivariable analysis.

**Table 3:** Multivariable-adjusted odds ratios of chronic kidney disease by Dichotomized^a^ Angiogenesis-related Factors

	Age, gender, and race-adjusted			Multivariable-adjusted^b^		
	Odds ratios	95% CI	*p*-value	Odds ratios	95% CI	*p*-value
VEGF-A ≥ 160.7 pg/mL	1.84	1.17–2.90	0.01	2.40	1.20–4.81	0.01
Angiopoietin-1 ≥ 5659.5 pg/mL	0.87	0.55–1.36	0.54	1.15	0.57–2.32	0.70
Angiopoietin-1/VEGF-A ≤ 24.2	3.19	2.00–5.10	< 0.001	3.59	1.80–7.18	0.0003
VEGFR-1 ≥ 159.7 ng/mL	1.10	0.70–1.75	0.68	1.36	0.67–2.75	0.40
VEGFR-2 ≥ 28.3 ng/ml	1.28	0.81–2.01	0.29	1.11	0.55–2.22	0.78
Pentraxin-3 ≥ 1.13 ng/mL	1.86	1.18–2.94	0.008	1.74	0.86–3.52	0.13

^a^Dichotomized as upper tertile compared to lower two tertiles for all biomarkers except the ratio of angiopoietin-1/VEGF-A, which was dichotomized as lowest tertile compared to upper two tertiles

^b^Adjusted for age, race, gender, current cigarette smoking, weekly alcohol consumption, physical activity ≥twice/week, BMI, LDL-cholesterol, HDL-cholesterol, C-reactive protein, fasting plasma glucose, systolic BP, use of aspirin or lipid-lowering, antihypertensive, or antidiabetic medications, and history of CVD

In multivariable adjusted logistic regression models stratified by diabetes status, strong but non-significant associations were observed for the highest tertile of VEGF-A (OR(95% CI) 6.47(0.89–47.1), *p* = 0.07), lowest tertile of the angiopoietin-1/VEGF-A ratio (OR (95% CI) 3.47(0.50–23.9), *p* = 0.21), and the highest tertile of PTX-3 (OR (95% CI) 8.03(0.93–69.6), *p* = 0.06) in diabetics (see Additional file 1: Table S1). Among non-diabetic subjects, a non-significant association was observed for the highest tertile of VEGF-A (OR (95% CI) 1.61 (0.89–2.92), *p* = 0.12), while significant associations were observed for the lowest tertile of the angiopoietin-1/VEGF-A ratio (OR (95% CI) 3.02 (1.64–5.57), *p* = 0.0004) and the highest tertile of PTX-3 (OR (95% CI) 1.98 (1.06–3.69), *p* = 0.03). No significant associations were observed for angiopoietin-1, VEGFR-1 or VEGFR-2 in multivariable adjusted models in diabetic or non-diabetic subjects.

In multivariable adjusted quantile regression models stratified by stage of CKD, median VEGF-A significantly increased with increasing severity of CKD (Table 4). Median angiopoietin-1/VEGF-A ratio significantly decreased with increasing CKD severity. PTX-3 increased with increasing CKD severity, though this did not achieve statistical significance. VEGFR-1 level increased significantly with increasing severity of CKD. Medians for angiopoietin-1 and VEGFR-2 did not differ by stage of CKD.

**Table 4:** Angiogenesis-related factors according to chronic kidney disease stage

	Multivariable-adjusted median (IQR)^a^			*p*-value
	Non-CKD controls (*N* = 201)	CKD Stage 3 (*N* = 142)	CKD Stage 4/5 (*N* = 59)	
VEGF-A, pg/mL	114.17 (72.45, 170.32)	128.71 (89.19, 214.49)	134.27 (95.73, 178.73)	0.001
Angiopoeitin-1, pg/mL	4270.5 (2763.7, 6537.2)	4026.3 (2511.6, 6881.9)	3753.5 (2423.0, 6181.4)	0.44
Angiopoeitin-1/VEGF-A	36.55 (25.71, 61.10)	26.36 (18.82, 50.19)	23.47 (16.83, 42.51)	0.001
VEGFR-1, ng/mL	144.16 (123.74, 168.05)	135.96 (118.60, 160.40)	163.96 (135.19, 184.76)	0.04
VEGFR-2, ng/mL	26.28 (23.10, 29.69)	25.78 (22.23, 29.68)	27.85 (23.12, 31.10)	0.19
Pentraxin-3, ng/mL	0.89 (0.58, 1.18)	1.01 (0.79, 1.38)	1.04 (0.78, 1.58)	0.06

^a^Adjusted for age, race, gender, current cigarette smoking, weekly alcohol consumption, physical activity ≥twice/week, BMI, LDL-cholesterol, HDL-cholesterol, C-reactive protein, fasting plasma glucose, systolic BP, use of aspirin or lipid-lowering, antihypertensive, or antidiabetic medications, and history of CVD

In multivariable adjusted polytomous logistic regression models, the association between high VEGF-A and CKD was observed for both stage 3 and 4/5 (OR (95% CI) 2.75(1.34–5.64) and 1.62(0.64–4.11), respectively, *p* = 0.02). The association between low angiopoietin-1/VEGF-A ratio and CKD was observed for both stage 3 and stage 4/5 (OR (95% CI) 3.07 (1.49–6.32) and 6.17 (2.48–15.3) respectively, *p* = 0.0003). A non-significant association between elevated PTX-3 and CKD was observed for both stage 3 and stage 4/5 CKD (OR (95% CI) 1.58(0.76–3.31) and 2.15(0.86–5.37), respectively, *p* = 0.2). No association was observed between high VEGFR-1 and stage 3 CKD, but a significant association was observed for stage 4/5 (OR (95% CI) 0.99(0.46–2.10) and 3.01(1.19–7.58), respectively, *p* = 0.02). No association was observed for angiopoietin-1 or VEGFR-2.

In the sensitivity analysis, medians of the biomarkers were compared for diabetic and non-diabetic cases. There was no significant difference in the medians of the biomarkers between the diabetic and non-diabetic CKD patients for angiopoietin-1, VEGF-A, angiopoietin-1/VEGF-A ratio, VEGFR-1, VEGFR-2, or pentraxin-3.

## Discussion

The present study indicated that higher VEGF-A and pentraxin-3 levels and a lower angiopoietin-1/VEGF-A ratio may be associated with increased risk of CKD. These associations remained after adjustment for established CKD risk factors as well as CVD and the use of antihypertensive, antidiabetic, lipid-lowering medications, and aspirin. These findings suggest that abnormal angiogenesis is present in patients with CKD.

Our study reports that plasma VEGF-A is significantly higher in patients with pre-dialysis CKD compared to controls. Animal and laboratory studies have suggested that increased VEGF-A expression causes glomerular hypertrophy, proliferation of podocytes, mesangial cell proliferation, extracellular matrix expansion, interstitial fibrosis, and proteinuria [34, 35]. The therapeutic effects of anti-VEGF-A and anti-angiogenic factors in experimental diabetic nephropathy have been reported, including amelioration of increases in urinary albumin excretion, glomerular volume, glomerular basement membrane thickening, in addition to decreased slit pore density and nephrin quantity [36–38]. Urinary VEGF was reported to be elevated in patients with diabetic nephropathy and positively associated with proteinuria [39]. Furthermore, plasma VEGF-A levels have previously been found to be associated with progression to ESRD in 67 patients with diabetic CKD [19]. However, Lindenmeyer et al. reported a decrease in mRNA expression of VEGF-A in the renal interstitium of patients with diabetic nephropathy in a small study [20]. A study of murine folic acid induced nephropathy found depleted VEGF-A in kidney tissue, but increased circulating VEGF-A, possibly from damage to the systemic vasculature induced by folic acid [17]. Our study findings support the hypothesis that increased circulating VEGF-A may be associated with increased risk of CKD. Further studies are warranted to examine the causal relationship of VEGF-A and the progression of CKD. Additionally, our study suggests that the treatment targeting VEGF in CKD needs further careful evaluation due to inconsistency between increased circulating VEGF-A and decreased renal expression of VEGF-A in findings from different studies.

Our study identified lower angiopoietin-1 in CKD patients than non-CKD controls, and lower angiopoietin-1 with increased CKD severity, though these differences did not achieve statistical significance. Decreased angiopoietin-1 has been reported in pre-dialysis CKD in children [25], but was not associated with eGFR [40] or mortality among patients with CKD [24]. Animal studies indicate that treatment with angiopoietin-1 might reduce kidney damage in unilateral ureteral obstruction, streptozotocin-induced type-1 diabetes, and folic acid induced nephropathy [15–17]. Deletion of angiopoietin-1 from mice embryos coupled with injury or microvascular stress caused organ damage, accelerated angiogenesis and fibrosis, suggesting angiopoietin-1 may balance the angio-fibrogenic response associated with elevated VEGF-A and angiopoietin-2 levels from tissue injury and microvascular disease, like that observed in diabetes [18]. More studies are warranted to confirm the relationship of angiopoietin-1 and CKD in humans.

A low ratio of angiopoietin-1 to VEGF-A was significantly associated with odds of CKD in our study. Similar associations were observed in both diabetic and non-diabetic subjects, but the associations achieved significance only in non-diabetic subjects likely due to the small sample size of the diabetic group. Lower angiopoietin-1, relative to VEGF-A concentrations, may be associated with impaired angiogenesis and enhanced endothelial leakage induced by VEGF-A as co-expression of angiopoietin-1 and VEGF-A enhances angiogenesis [19] and angiopoietin-1 can potently block VEGF-induced endothelial permeability in vitro [41]. Podocyte specific repletion of angiopoietin-1 in a model of type 1 diabetes decreased glomerular endothelial cell proliferation, hyperfiltration and albuminuria by 70% [16]. Angiopoietin-1 deficiency and VEGF-A excess is thought to destabilize endothelia in type 1 diabetic mice, and the improvements observed in mice treated with angiopoietin-1 may be attributable to vascular stabilization from attenuation of VEGF-A signaling by increased angiopoietin-1 [16, 18]. Our study sample was found to have a similar growth factor milieu, with angiopoietin-1 deficiency relative to excess VEGF-A among CKD patients. A recent study suggested that low angiopoietin-1 level was positively associated with abnormal cardiac structure in stages 3–5 CKD patients [42]. Further studies are warranted to investigate whether imbalanced angiopoietin-1 and VEGF-A may be associated with an increased risk of ESRD and CVD in CKD patients.

The VEGFR-1 and VEGFR-2 levels were not significantly different between CKD patients and controls. VEGFR-1 was significantly elevated in subjects with stage 4 and 5 CKD compared to the controls, suggesting that high VEGFR-1 may be associated with more severe CKD, which may conform with the observation that elevated VEGFR-1 was associated with inflammation and mortality in dialysis patients in previous studies [21, 22]. Unlike our study, a previous study reported VEGFR-2 to be lower in dialysis patients [22]. The underlying explanation for this inconsistency between our study finding and the other’s may be due to differences in the study population and the severity of CKD.

Pentraxin-3 levels were significantly higher in patients with CKD compared to the controls after adjusting for multiple confounding factors including C-reactive protein in our study, even though pentraxin-3 did not increase substantially with increased severity of CKD and the odds of CKD associated with pentraxin-3 did not achieve statistical significance in multivariable logistic analysis. When logistic regression models were run separately in diabetic and non-diabetic subjects, a statistically significant doubling of odds of CKD among those with high pentraxin-3 was observed in non-diabetic subjects, while a much larger but not statistically significant increase in odds of CKD was observed among diabetic subjects. The inconsistency of these findings is likely due to limited statistical power in the subgroup analyses. Pentraxin-3 has been associated with endothelial dysfunction, decreased eGFR, and proteinuria in previous studies [43, 44]. It is also associated with inflammation in cardiovascular disease [45, 46]. However, the association observed between PTX-3 and CKD is independent of inflammatory and endothelial dysfunction biomarker C-reactive protein in our study, suggesting that PTX-3 might play a role in pathogenesis of CKD via an additional pathway such as abnormal angiogenesis. Future study is needed in this important area.

There are several noteworthy strengths of this study. Our study is among the largest of early studies that have found plasma VEGF-A levels were significantly increased in patients with CKD and that there might be a significant imbalance of VEGF-A and angiopoetin-1 in CKD patients. These associations were independent of multiple covariables for CKD that were carefully measured in our study. Furthermore, our study included a racially diverse group of patients with variable degrees of renal function. This study also had several limitations. First, this is a cross-sectional analysis, which prevents the determination of direction of the relationship between these angiogenic factors and CKD. Second, our study has a relatively small sample size. There is limited statistical power to do subgroup analyses by severity of CKD and diabetes status. Third, the majority of our CKD cases have stage 3 CKD, with only a minority in stage 4 or 5. An underrepresentation of more severe CKD may have limited our ability to identify associations between biomarkers and severe CKD. A larger prospective cohort study might provide more definitive evidence for the association of plasma angiogenic factors with CKD.

## Conclusions

In conclusion, this study shows that higher circulating VEGF-A and pentraxin-3 levels as well as a lower angiopoetin-1/VEGF-A ratio may associated with an increased risk of CKD. Future prospective studies are warranted to examine whether these angiogenic factors play a role in progression of CKD and these abnormalities of angiogenic factors may serve as therapeutic targets for treatment of CKD.

## Additional file

Additional file 1: **Table S1.** Age, Race and Gender Adjusted and Multivariable-adjusted Odds Ratios of Chronic Kidney Disease in Patients with and without Diabetes by Dichotomized* Angiogenesis-related Factors. The data presented in the table describe the associations between the angiogenesis related factors and CKD in diabetics and non-diabetics. (DOCX 16 kb)

## Abbreviations

BMI: Body mass index
BP: Blood pressure
CKD: Chronic kidney disease
CVD: Cardiovascular disease
eGFR: estimated glomerular filtration rate
ESRD: End-stage renal disease
FGF: Fibroblast growth factor
SCr: Serum creatinine
sVEGFR: soluble VEGF receptor
VEGF: Vascular endothelial growth factor
VEGFR: Vascular endothelial growth factor receptor

## References

[1] Coresh J, Selvin E, Stevens LA, Manzi J, Kusek JW, Eggers P (2007). Prevalence of chronic kidney disease in the United States. JAMA.

[2] Mills KT, Xu Y, Zhang W, Bundy JD, Chen CS, Kelly TN (2015). A systematic analysis of worldwide population-based data on the global burden of chronic kidney disease in 2010. Kidney Int.

[3] Matsushita K, van der Velde M, Astor BC, Woodward M, Levey AS, Chronic Kidney Disease Prognosis C (2010). Association of estimated glomerular filtration rate and albuminuria with all-cause and cardiovascular mortality in general population cohorts: A collaborative meta-analysis. Lancet.

[4] Coresh J, Turin TC, Matsushita K, Sang Y, Ballew SH, Appel LJ (2014). Decline in estimated glomerular filtration rate and subsequent risk of end-stage renal disease and mortality. JAMA.

[5] Levey AS, Coresh J (2012). Chronic kidney disease. Lancet.

[6] Wong MG, Pollock CA (2014). Biomarkers in kidney fibrosis: Are they useful?. Kidney Int Suppl (2011).

[7] Zoja C, Zanchi C, Benigni A (2015). Key pathways in renal disease progression of experimental diabetes. Nephrol Dial Transplant.

[8] Cooper ME, Vranes D, Youssef S, Stacker SA, Cox AJ, Rizkalla B (1999). Increased renal expression of vascular endothelial growth factor (VEGF) and its receptor VEGFR-2 in experimental diabetes. Diabetes.

[9] Hara A, Wada T, Furuichi K, Sakai N, Kawachi H, Shimizu F (2006). Blockade of VEGF accelerates proteinuria, via decrease in nephrin expression in rat crescentic glomerulonephritis. Kidney Int.

[10] Kang DH, Anderson S, Kim YG, Mazzalli M, Suga S, Jefferson JA (2001). Impaired angiogenesis in the aging kidney: Vascular endothelial growth factor and thrombospondin-1 in renal disease. Am J Kidney Dis.

[11] Kang DH, Joly AH, Oh SW, Hugo C, Kerjaschki D, Gordon KL (2001). Impaired angiogenesis in the remnant kidney model: I. Potential role of vascular endothelial growth factor and thrombospondin-1. J Am Soc Nephrol.

[12] Long DA, Woolf AS, Suda T, Yuan HT (2001). Increased renal angiopoietin-1 expression in folic acid-induced nephrotoxicity in mice. J Am Soc Nephrol.

[13] Rizkalla B, Forbes JM, Cao Z, Boner G, Cooper ME (2005). Temporal renal expression of angiogenic growth factors and their receptors in experimental diabetes: Role of the renin-angiotensin system. J Hypertens.

[14] Yuan HT, Tipping PG, Li XZ, Long DA, Woolf AS (2002). Angiopoietin correlates with glomerular capillary loss in anti-glomerular basement membrane glomerulonephritis. Kidney Int.

[15] Kim W, Moon SO, Lee SY, Jang KY, Cho CH, Koh GY (2006). COMP-angiopoietin-1 ameliorates renal fibrosis in a unilateral ureteral obstruction model. J Am Soc Nephrol.

[16] Dessapt-Baradez C, Woolf AS, White KE, Pan J, Huang JL, Hayward AA (2014). Targeted glomerular angiopoietin-1 therapy for early diabetic kidney disease. J Am Soc Nephrol.

[17] Long DA, Price KL, Ioffe E, Gannon CM, Gnudi L, White KE (2008). Angiopoietin-1 therapy enhances fibrosis and inflammation following folic acid-induced acute renal injury. Kidney Int.

[18] Jeansson M, Gawlik A, Anderson G, Li C, Kerjaschki D, Henkelman M (2011). Angiopoietin-1 is essential in mouse vasculature during development and in response to injury. J Clin Invest.

[19] Agarwal R, Duffin KL, Laska DA, Voelker JR, Breyer MD, Mitchell PG (2014). A prospective study of multiple protein biomarkers to predict progression in diabetic chronic kidney disease. Nephrol Dial Transplant.

[20] Lindenmeyer MT, Kretzler M, Boucherot A, Berra S, Yasuda Y, Henger A (2007). Interstitial vascular rarefaction and reduced VEGF-A expression in human diabetic nephropathy. J Am Soc Nephrol.

[21] Guo Q, Carrero JJ, Yu X, Barany P, Qureshi AR, Eriksson M (2009). Associations of VEGF and its receptors sVEGFR-1 and -2 with cardiovascular disease and survival in prevalent haemodialysis patients. Nephrol Dial Transplant.

[22] Yuan J, Guo Q, Qureshi AR, Anderstam B, Eriksson M, Heimburger O (2013). Circulating vascular endothelial growth factor (VEGF) and its soluble receptor 1 (sVEGFR-1) are associated with inflammation and mortality in incident dialysis patients. Nephrol Dial Transplant.

[23] Carmeliet P (2003). Angiogenesis in health and disease. Nat Med.

[24] David S, John SG, Jefferies HJ, Sigrist MK, Kumpers P, Kielstein JT (2012). Angiopoietin-2 levels predict mortality in CKD patients. Nephrol Dial Transplant.

[25] Shroff RC, Price KL, Kolatsi-Joannou M, Todd AF, Wells D, Deanfield J (2013). Circulating angiopoietin-2 is a marker for early cardiovascular disease in children on chronic dialysis. PLoS One.

[26] Leali D, Inforzato A, Ronca R, Bianchi R, Belleri M, Coltrini D (2012). Long pentraxin 3/tumor necrosis factor-stimulated gene-6 interaction: A biological rheostat for fibroblast growth factor 2-mediated angiogenesis. Arterioscler Thromb Vasc Biol.

[27] Pickering TG, Hall JE, Appel LJ, Falkner BE, Graves J, Hill MN (2005). Recommendations for blood pressure measurement in humans and experimental animals: Part 1: Blood pressure measurement in humans: A statement for professionals from the Subcommittee of Professional and Public Education of the American Heart Association Council on high blood pressure research. Circulation.

[28] Becker A, Befus A, Graham C, HayGlass K, Lotoski L, Lopez P (2017). Stability of pro-and anti-inflammatory immune biomarkers for human cohort studies. J Transl Med.

[29] Shabihkhani M, Lucey GM, Wei B, Mareninov S, Lou JJ, Vinters HV (2014). The procurement, storage, and quality assurance of frozen blood and tissue biospecimens in pathology, biorepository, and biobank settings. Clin Biochem.

[30] Lee J-E, Kim SY, Shin S-Y (2015). Effect of repeated freezing and thawing on biomarker stability in plasma and serum samples. Osong Public Health Res Perspect.

[31] Levey AS, Stevens LA, Schmid CH, Zhang YL, Castro AF, Feldman HI (2009). A new equation to estimate glomerular filtration rate. Ann Intern Med.

[32] LaVange LM, Koch GG (2006). Rank score tests. Circulation.

[33] McGreevy KM, Lipsitz SR, Linder JA, Rimm E, Hoel DG (2009). Using median regression to obtain adjusted estimates of central tendency for skewed laboratory and epidemiologic data. Clin Chem.

[34] Farris AB, Colvin RB (2012). Renal interstitial fibrosis: Mechanisms and evaluation. Curr Opin Nephrol Hypertens.

[35] Liu E, Morimoto M, Kitajima S, Koike T, Yu Y, Shiiki H (2007). Increased expression of vascular endothelial growth factor in kidney leads to progressive impairment of glomerular functions. Journal of the American Society of Nephrology : JASN..

[36] de Vriese AS, Tilton RG, Elger M, Stephan CC, Kriz W, Lameire NH (2001). Antibodies against vascular endothelial growth factor improve early renal dysfunction in experimental diabetes. J Am Soc Nephrol.

[37] Flyvbjerg A, Dagnaes-Hansen F, De Vriese AS, Schrijvers BF, Tilton RG, Rasch R (2002). Amelioration of long-term renal changes in obese type 2 diabetic mice by a neutralizing vascular endothelial growth factor antibody. Diabetes.

[38] Sung SH, Ziyadeh FN, Wang A, Pyagay PE, Kanwar YS, Chen S (2006). Blockade of vascular endothelial growth factor signaling ameliorates diabetic albuminuria in mice. J Am Soc Nephrol.

[39] Kim NH, Oh JH, Seo JA, Lee KW, Kim SG, Choi KM (2005). Vascular endothelial growth factor (VEGF) and soluble VEGF receptor FLT-1 in diabetic nephropathy. Kidney Int.

[40] Chang FC, Chiang WC, Tsai MH, Chou YH, Pan SY, Chang YT (2014). Angiopoietin-2-induced arterial stiffness in CKD. J Am Soc Nephrol.

[41] Gamble JR, Drew J, Trezise L, Underwood A, Parsons M, Kasminkas L (2000). Angiopoietin-1 is an antipermeability and anti-inflammatory agent in vitro and targets cell junctions. Circ Res.

[42] Tsai YC, Lee CS, Chiu YW, Kuo HT, Lee SC, Hwang SJ (2016). Angiopoietin-2, Angiopoietin-1 and subclinical cardiovascular disease in chronic kidney disease. Sci Rep.

[43] Dubin R, Shlipak M, Li Y, Ix J, de Boer IH, Jenny N (2011). Racial differences in the association of pentraxin-3 with kidney dysfunction: The multi-ethnic study of atherosclerosis. Nephrol Dial Transplant.

[44] Suliman ME, Yilmaz MI, Carrero JJ, Qureshi AR, Saglam M, Ipcioglu OM (2008). Novel links between the long pentraxin 3, endothelial dysfunction, and albuminuria in early and advanced chronic kidney disease. Clin J Am Soc Nephrol.

[45] Inoue K, Kodama T, Daida H (2012). Pentraxin 3: A novel biomarker for inflammatory cardiovascular disease. Int J Vasc Med.

[46] Latini R, Maggioni AP, Peri G, Gonzini L, Lucci D, Mocarelli P (2004). Prognostic significance of the long pentraxin PTX3 in acute myocardial infarction. Circulation.

