# Supplementary material for: The association of angiogenic factors and chronic kidney disease
Source: BMC Nephrol. 2018 May 21;19:117. doi: 10.1186/s12882-018-0909-2 (PMC5963107; doi:10.1186/s12882-018-0909-2)
Supplement: Supplementary file 1 — Table S1. Age, Race and Gender Adjusted and Multivariable-adjusted Odds Ratios of Chronic Kidney Disease in Patients with and without Diabetes by Dichotomized* Angiogenesis-related Factors. The data presented in the table describe the associations between the angiogenesis related factors and CKD in diabetics and non-diabetics. (DOCX 16 kb) [file 12882_2018_909_MOESM1_ESM.docx]

**Table S1. Age, Race and Gender Adjusted and Multivariable-adjusted Odds Ratios of Chronic Kidney Disease in Patients with and without Diabetes by Dichotomized* Angiogenesis-related Factors.**

|  |  | Diabetic Subjects | | | | | | |
| --- | --- | --- | --- | --- | --- | --- | --- | --- |
|  |  | N=110 | | | | | | |
|  |  | Age, Race and Gender Adjusted | | |  | Multivariable Adjusted† | | |
|  |  | Odds Ratio | 95% CI | P-value |  | Odds Ratio | 95% CI | p-value |
| VEGF-A ≥ 160.7 pg/mL |  | 4.63 | 0.94-22.9 | 0.06 |  | 6.47 | 0.89-47.1 | 0.07 |
| Angiopoietin-1 ≥ 5659.5 pg/mL |  | 1.87 | 0.46-7.69 | 0.38 |  | 1.87 | 0.26-13.7 | 0.54 |
| Angiopoietin-1/VEGF-A ≤ 24.2 |  | 2.91 | 0.73-11.7 | 0.13 |  | 3.47 | 0.50-23.9 | 0.21 |
| VEGFR-1 ≥ 159.7 ng/mL |  | 0.87 | 0.25-3.01 | 0.83 |  | 1.05 | 0.19-5.68 | 0.96 |
| VEGFR-2 ≥ 28.3 ng/ml |  | 0.87 | 0.26-2.89 | 0.82 |  | 2.63 | 0.47-14.9 | 0.27 |
| Pentraxin-3 ≥ 1.13 ng/mL |  | 4.98 | 1.00-24.8 | 0.05 |  | 8.03 | 0.93-69.6 | 0.06 |
|  |  | Non-diabetic Subjects | | | | | | |
|  |  | N=292 | | | | | | |
|  |  | Age, Race and Gender Adjusted | | |  | Multivariable Adjusted† | | |
|  |  | Odds Ratio | 95% CI | P-value |  | Odds Ratio | 95% CI | p-value |
| VEGF-A ≥ 160.7 pg/mL |  | 1.55 | 0.91-2.64 | 0.11 |  | 1.61 | 0.89-2.92 | 0.11 |
| Angiopoietin-1 ≥ 5659.5 pg/mL |  | 0.87 | 0.51-1.48 | 0.60 |  | 1.10 | 0.60-1.99 | 0.76 |
| Angiopoietin-1/VEGF-A ≤ 24.2 |  | 3.18 | 1.85-5.47 | <0.0001 |  | 3.02 | 1.64-5.57 | 0.0004 |
| VEGFR-1 ≥ 159.7 ng/mL |  | 1.14 | 0.66-1.97 | 0.64 |  | 1.09 | 0.58-2.03 | 0.79 |
| VEGFR-2 ≥ 28.3 ng/ml |  | 1.28 | 0.75-2.20 | 0.37 |  | 1.60 | 0.87-2.93 | 0.13 |
| Pentraxin-3 ≥ 1.13 ng/mL |  | 1.60 | 0.93-2.75 | 0.09 |  | 1.98 | 1.06-3.69 | 0.03 |

* Dichotomized as upper tertile compared to lower two tertiles for all biomarkers except the ratio of angiopoietin-1/VEGF-A, which was dichotomized as lowest tertile compared to upper two tertiles, and Pentraxin-3 which was dichotomized at the median.

† Adjusted for age, race, gender, current cigarette smoking, weekly alcohol consumption, physical activity ≥twice/week, BMI, LDL-cholesterol, HDL-cholesterol, C-reactive protein, systolic BP, and history of CVD.
